# Editorial: Insect physiological changes during insect-plant interaction

**DOI:** 10.3389/fphys.2023.1175813

**Published:** 2023-03-08

**Authors:** Wenwu Zhou, Xiaofeng Xia, Artemio Mendoza-Mendoza, Waqas Wakil, Komivi Senyo Akutse, Xiaoli Bing

**Affiliations:** ^1^ State Key Laboratory of Rice Biology, Ministry of Agricultural and Rural Affairs Key Laboratory of Molecular Biology of Crop Pathogens and Insect Pests, Institute of Insect Sciences, Zhejiang University, Hangzhou, China; ^2^ State Key Laboratory of Ecological Pest Control for Fujian and Taiwan Crops, Institute of Applied Ecology, Fujian Agriculture and Forestry University, Fuzhou, China; ^3^ Faculty of Agriculture and Life Sciences, Department of Wine, Food and Molecular Biosciences, Lincoln University, Lincoln, New Zealand; ^4^ Department of Entomology, University of Agriculture, Faisalabad, Pakistan; ^5^ Senckenberg Deutsche Entomologische Institute (SDEI), Müncheberg, Germany; ^6^ International Centre of Insect Physiology and Ecology (icipe), Nairobi, Kenya; ^7^ Department of Entomology, Nanjing Agricultural University, Nanjing, China

**Keywords:** insect-plant interaction, insect physiology, plant defense, herbivores, microorganisms

The interactions between phytophagous insects and their host plants result from a long and continuous evolutionary process (Beran and Petschenka, 2022). Such ecological relationships led to an extraordinary diversity of insects and shaped their complex physiological systems (Wheat et al., 2007). The impacts of host plants on the physiology of herbivorous insects have increasingly become a paramount focus that should not be ignored. Chemical compounds’ composition of plants have not only significant variations in the inter/intra species aspect but also show spatiotemporal variations in different developmental stages and tissue types, or under changeable environments in nature, which lead to the resource assimilation and fitness challenges of insects (Delucia et al., 2012; Brütting et al., 2017). These close interations with plants affect the ecological plasticity of the performance of insect herbivores (Barker et al., 2019). Currently, in-depth exploration of the host plants’ effect on insects has become a research hotspot of insect physiology, however to test the highly complex hypothesis can be difficult. The current Research Topic aimed to highlight the recent developments on 1) how physiological changes occurred in herbivores during their interaction with host plants, 2) how these physiological changes in insects could be affected by other biotic factors.

The interaction between the diamond back moth (DBM), *Plutella xylostella* (Linnaeus) (Lepidoptera: Plutellidae), and the crucifer plants is one of the most well-known models in chemical ecology. It was established that the β-glucosidases (myrosinase) and glucosinolates (GSLs) are stored in distinct subcellular compartments in plants, and the contact between them due to herbivory damage can rapidly produce a group of toxic aglycones, such as isothiocyanates (ITCs) (Textor and Gershenzon, 2009). These sulfur-containing aglycones can be deterrent to many insect pests but not to DBM, since DBM can hydrolyze the glucosinolates into desulfur-glucosinolates by using glucosinolate sulfatases (GSSs) (Winde and Wittstock, 2011). Chen et al. identified 13 glycoside hydrolase family 1 (GH1) genes in DBM. Among them, the midgut-specific gene *Px008848* is induced by feeding on the host plant. *In vitro* expression of Px008848 protein showed β-glucosidase activity, and *Arabidopsis thaliana* leaves treated with this protein can significantly reduce the survival of DBM larvae. Meanwhile, knocking out this gene by CRISPR/Cas9 enhanced the survival rate of this insect on *A. thaliana*, indicating that this gene might be involved in the interaction between DBM and their host plants; and consequently illuminating our understanding of the evolutionary function of this gene family in the insect-plant interaction.

While studies suggested that several aboveground feeding herbivores have developed physiological strategies to overcome the GSL-ITC plant defenses (Crespo et al., 2012), how belowground herbivores deal with these toxic secondary compounds remains unclear. Sontowski et al. investigated two underground *Brassica* specialists, *Delia radicum* (Linnaeus) and *D*. *floralis* (Fallén) (Diptera: Anthomyiidae) to analyze the way they detoxify the GSL-ITC defenses of their host plants. Both species detoxify ITCs by different enzymatic processes, where ITCs negatively affected their performance/survival, and even at the gene level, they exhibited varying response following their exposure to ITC. The same detoxification mechanisms could be related to fact that the cabbage root fly *D*. *radicum* prefer Chinese cabbage than broccoli (Lamy et al., 2018).

The fall armyworm, *Spodoptera frugiperda* (J.E. Smith) (Lepidoptera: Noctuidae), is a polyphagous lepidopteran pest. Zheng et al. evaluated the impacts of two host plants (corn and goosegrass) on the development and reproduction of *S. frugiperda*. They found that females fed on goosegrass had shorter ovarioles and laid fewer eggs than those fed on corn. Transcriptomic analysis revealed that 881 genes involved in ovarian development were differentially expressed when fed on corn and goosegrass. Among them, the juvenile hormone biosynthetic genes, 20-hydroxyecdysone biosynthetic genes, and ovary-relevant functional genes were differentially expressed in the ovary of the *S. frugiperda* after feeding on goosegrass. It would be interesting to find out further how host plant type and feeding preference affect ovarian development in *S. frugiperda*.

Insects rely on metabolic enzymes to detoxify the secondary metabolites in the host plant tissues they consume. Glutathione-S-transferases (GSTs) are among herbivores’ most well-studied metabolic enzymes (Rane et al., 2019). Venthur et al. identified 22 GSTs in the greater wax moth, *Galleria mellonella* (Linnaeus) (Lepidoptera: Pyralidae), a global pest for beehives. Treating the larvae with root extract of *Berberis microphylla* G. Forst (Ranunculales: Berberidaceae), as well as the alkaloids from these extracts, the alkaloids berberine and palmatine were found to induce the accumulation of transcripts for some of these GSTs. The protein structure prediction suggested putative interactions between these GSTs and chemicals.

Pathogens can affect the performance of vector insects either by direct infection or indirectly changing host plants’ physiological status (Colvin et al., 2006). Citrus Huanglongbing disease is a destructive disease caused by the pathogen *Candidatus* Liberibacter asiaticus (*C*Las), and the Asian citrus psyllid *Diaphorina citri* Kuwayama (Hemiptera: Liviidae) is a key vector for this pathogen (Galdeano et al., 2020). Zhang et al. found that *C*Las can change the abundance, composition and utilization efficiency of different amino acids in citrus host plants, thus affecting the construction of amino acids in nymphs and adults of *D*. *citri*, which could potentially affect their transmission of *C*Las. This study provided relevant evidence that pathogen-mediated changes of primary metabolites in plants can affect the performance of their insect vector.
